# The Physiology of Epilepsy and of Paroxysmal Apoplexy, Paralysis, Mania, &c.

**Published:** 1853-07

**Authors:** Marshall Hall


THE NORTH-WESTERN

MEDICAL AND SURGICAL JOURNAL.

NEW SERIES.

VOL II. JULY, 1853. No. 3.

### ORIGINAL COMMUNICATIONS.

"*Les symptomes de l'epilepsie sont tellement extraordinaires, tellement au dessus de toute explication physiologique ; les causes de cette maladie sont tellement inconnues, que,*" *&c.* ESQUIROL.

"*I think no disease is better understood in its physiology or pathology, since the detection of the* SPINAL SYSTEM, *than Epilepsy.*

THE AUTHOR.

ART. I.—*The Physiology of Epilepsy, and of Paroxysmal Apoplexy, Paralysis, Mania, &c.* By MARSHALL HALL, M.D., F.R.S., ETC.

I HAVE frequently used the subjoined *Table* as the heads of a lecture on the important subject of this communication. I begin by presenting it to your readers :—

### I. *General View of Epilepsia, Apoplexy, Paralysis, and Mania.*

1. Epilepsy,
2. Apoplexy,
3. Paralysis,
4. Mania,—all which may be different phases of one disease—may each be arranged into—

CLASS I. The Paroxysmal—
1. Of inorganic Origin ;
2. Of Recurrent Paroxysmal Form ;
3. Of short Duration, terminating in perfect recovery, or
4. Fatal, without post-mortem appearances, or
5. With such as are Effects only.

CLASS II. The Permanent—
1. Of Organic Origin ;
2. Of Permanent Form.

### II. *Inorganic Epilepsy and Apoplexy are caused by*

**I. Acting through the Diastaltic Spinal System—**

**II. Upon the Muscular and Venous Systems of the Neck, whence,—****

| Causes. | I. The Mode of Action. | II. The Medium of Action. | I. Spasmodic Action— of | I. Trachelismus, with— |
|---|---|---|---|---|
| I. The Emotions.— Excitement, Anger, Fright; Awaking, &c. II. Certain Physical Causes— 1. Posture; Effort— Mental or Physical; Fatigue; &c. 2. Hyperhœmia; Anœmia; Cachœmia; &c. | Catastaltic or Direct— through* | II. Exodic Nerves, viz. 1. The Descendens Facialis.— 2. The Descendens Myoglossalis— 3. The Spinal Accessory— 4. The Spinal— (The Pneumogastric, with Palpitation, &c. | 1. The Platysma-myoid— 2. The Cleido-mastoid, the Omo-hyoid, &c.— 3. The Trapezius, the Scaleni, &c.— 4. The Subclavian— | Compression of 1. The External Jugular— 2. The Internal Jugular— 3. The Vertebral— 4. The Subclavian—Veins, or Phlebismus—with Congestion; Effusion; Softening? Obstructed Arteries? &c. |
| III. The Sleep— | Autostaltic,—in* | 1. Recurrent Laryngeal— 2. The Intercostal— 3. The Abdominal— | 1 The Arytænoid— 2. The Intercostal— 3. The Abdominal— | II. Laryngismus. 1. Incomplete—with Stridor; &c. 2. Complete—with Efforts of Expiration, or Dyspnœa, &c. |
| IV. The Irritations— 1. Dental— 2. Gastric— 3. Intestinal— 4. Uterine— 1. Catamenial— 2. Puerperal— | Diastaltic or Reflex,— through I. Eisodic Nerves, viz. 1. The Trifacial— 2. The Pneumogastric— 3. The Spinal—* | | | |
| V. Venus nimia præsertim solitaria. | | | | |

### III. *Inorganic Epilepsy, which may be divided into the—*

II. **Irritation of the Medulla Oblongata, and Congestions of the Cerebrum—

I. Epilepsia Mitior, and this into

I. Epilepsia Evanescens, with

I. 1. Obscure Trachelismus;

2. Oblivium, Confusion, Vertigo; &c.

3. Distortion of the Eyes, Features, Fingers, &c., &c.

4, Nutatio; Falling; &c.

5. Faintishness;

6. Oneirodynia.

II. Epilepsia Trachelea, involving the Spinal Accessory, &c., with

1. Aura; Flocci; Tinnitus; Odor Moschi, &c.

II. 2. Manifest Trachelismus—Fixed Head, Torticollis, Obstipitas;

3. Spasmodic Aura?

4. Laryngeal, Stridor, Cry, Dyspnœa;

5. Bitten Tongue, Lip, or Cheek;

6. Foam, Rattles, &c.

1. Flushing;
2. Stupor;
3. Falling with Violence;

II. Epilepsia Gravior, and this into

I. Epilepsia Laryngea, involving the superior Laryngeal, &c., with

I Trachelismus;
1. Purpurescence;
2. Stupor; Coma;

II. Spasmodic Laryngismus,

1. Dysecpnœa,

2. Convulsion leading to

1. Coma,

2. Stertor with VIII. II. II.

3. Augmented Coma;

4. Mania,

5. Amentia,

6. Spasmo-Paralysis.

7, Death.

} Superseded by Tracheotomy, precisely in proportion to the Laringismus and its Dyspnœa; leaving, at the most, an Epilepsia Abortiva,

II. Epilepsia Syncopalis, with
1. Pallor, Lividity;
2. Syncope;
3. Sudden Death.

Hidden Seizures;—Mania?—Crime?—Punishment!

Augmented Spinal Excitability and Susceptibility to Attacks.

The Treatment should be equally free from Empiricism in both and consist in—
I. Avoiding the exciting Causes.
II. Giving
1. Antacids.
2. Gentle Emetics?
3. Gentle but efficient Aperients,
III. Attending strictly to Posture.
IV. Subduing Excitement by Hyoscyamus.
V. Subduing induced Susceptibility by Strychnia.
VI. Removing organic Effects by Mercurials.
VII. Restoring the general Health, by Air & Exercise, etc.,
and be founded on an adequate DIAGNOSIS,
Are there any Specifics?

### IV. *Inorganic Apoplexy which may be divided into the—*

II. **Congestion of the Cerebrum, and—

I. Apoplexia Mitior, and this into

I. Apoplexia Evanescens, with

1. Vertigo; Confusion;
2. Paralysis of Speech; of the Fingers of the Side; &c., &c.

II. Apoplexia Trachelea, with

I. Trachilismus, denoted by—
1. Flushing, Purpurescence, and
2. Tumidity, of the Face and Neck;
3, Vertigo; &c.
4. Stupor; &c., &c.
5. Falling;
6. Paroxysmal Paralysis,

II. Apoplexia Gravior, or

Apoplexia Laryngea; viz.

I. Trachilismus;
1. Purpurescence;
2. Cerebral Congestion;
3. Stupor; Coma;

II. Paralytic Laryngismus, with
4. Stertor; from Paralysis by counter-pressure of
 1. The Medulla Oblongata, and thence of
 2. The Pneumogastric, and especially of
  1. The Recurrents; with
5. Augmented Coma; of
  2. The Pharyngeals, with Dysphagia; of
  3. The Bronchial, with Rales, &c.
  4. The Cardiac,
  5. The Gastric, with correlative Derangements;
6. Death.

Augmented Spinal Excitability and Susceptibility to Attacks.

The Treatment should be equally free from Empiricism in both, and consist in—

I. Avoiding the exciting Causes.
II. Giving
 1. Antacids,
 2. Gentle Emetics?
 3. Gentle but efficient Aperients.
III. Attending strictly to Posture.
IV. Subduing Excitement by Hyoscyamus.
V. Subduing induced Susceptibility by Strychnia.
VI. Removing organic Effects by Mercurials.
VII. Restoring the general Health, by Air and Exercise, &c., and be founded on an adequate DIAGNOSIS.

Are there any Specifics?

A severe attack of Epilepsy too frequently terminates in *apoplectic* coma and stertor, and sometimes, though less frequently, in a paroxysm of *mania*; whilst repeated attacks lead to loss memory and of intellect in general, or *amentia*. *Paralysis*, partial or hemiplegic, is also a frequent result of the same dire disease. In these cases it is obvious that these different affections are but different phases of *one and the same* disease.

But each of these forms of malady may occur alone and distinctly, and constitute the sole disease. Are they not then, too, but different forms of one and the same pathological condition,—differing probably only in the specific part of the nervous centres primarily or secondarily affected? This opinion is, at least, the result of my own long-continued observation.

The *causes* of these seizures are enumerated in the *first* column of the *second* part of the table. This enumeration is followed by the *mode* and *media* of their action in the second, third, and fourth columns. The anatomist and physiologist will find in these an ample subject of meditation and study. The anatomy and the physiology of the *spinal system* must be previously well mastered, for these are, to diseases of the nervous system, what the stethoscope is to those of the lungs and the heart—the source of their *diagnosis*.

In the *fifth* and *sixth* columns, I have arranged the *muscles* which are chiefly excited into action, and the *veins* which are chiefly compressed by their action—whence result the special forms of the disease. These, too, must be well and deliberately considered by the scientific physician; for on these depend the spinal or epileptic, or the cerebral or apoplectic, forms of the disease; compression of the vertebral veins will be more likely to produce the former, that of the jugulars, the latter.

The contraction of the muscles may be limited to those of *the Neck*, the most familiar and marked form of which is *torticollis*; or it may involve those of the *larynx*, constituting *laryngismus*, so familiar also to every physician. In the former case the symptoms are generally less severe than in the latter; they may indeed be very slight; but they may also be formidable enough: every kind of contortion of the features, neck, limbs and trunk, with bitten tongue, may occur, with '*falling*,' &c., &c. But in the latter case, or laryngismus, the symptoms are more fearful still: the face and neck become tumefied and purpurescent, with asphyxia, apoplexy, &c., &c. The results of either, if repeated, may issue in impaired intellect. The apoplexy may be so profound as to lead to *stertor*, another form of laryngismus, but *paralytic* in its nature, that formerly noticed being *spasmodic.* Both may be imitated: a drop of water or a crumb of bread fallen on the rima glottidis will produce the *spasmodic* laryngismus; the division of the recurrent branches of the pneumogastric nerves, produces the paralytic.

It is only necessary to observe well to perceive how either form of laryngismus may involve the safety of the patient. It is in this case that trachectomy or rather tracheo*tony* steps in and presents us with the *presens remedium.* By this operation the disease is at once disarmed of its violence, and the result may be the preservation of life and even the restoration to health.

I beg here to adduce the case of Dr. Neill, of the Pennsylvania Hospital, Philadelphia, known to the profession through the medium of the American Journal of the Medical Sciences, so ably conducted by Dr. Hayes. I have also great pleasure in quoting a case by Dr. Herrick, of Chicago: The patient was brought into the U. S. Marine Hospital affected with violent epileptic fits, recurrent every twenty minutes, with spasmodic laryngismus, each leaving deeper and deeper coma and stertor, or paralytic laryngismus. It was obvious that the patient would speedily die of laryngeal dyspnœa, asphyxia and apoplexy. It was equally obvious that tracheotomy afforded a chance, the only chance, for life. Dr. Herrick did not hesitate to perform this operation. The patient was greatly relieved; the tumefaction of the face and neck, the stertorous dyspnœa, subsided; a degree of consciousness returned; there was a ray of hope that life might be preserved! But the bronchial tubes had become filled with mucus, a not unusual effect of severe epileptic seizures, and though rescued from laryngeal asphyxia, the patient died, and, as usual, rather suddenly, of bronchial asphyxia. If the operation had been performed earlier, that is before the excessive effusion of bronchial mucus had taken place, the patient might probably have been saved.

Dr. Herrick's conclusion from this case is most favorable to the operation. He would have recourse to it again under similar circumstances, and *earlier* if the opportunity were afforded him.

I can only add that the remaining columns of my table must also be carefully, most carefully studied, and especially that which treats of the influence of the pneumogastric nerve, in its laryngeal, bronchial, cardiac, pharyngeal and gastric branches, on the functions of the larynx, bronchia, heart, pharynx, stomach, especially and distinctly, in epileptic and apopletic affections. It presents a new field of inquiry for the scientific, anatomical and physiological physician. It presents a noble example of the superiority of science over empiricism, and the kind of study by which our profession is to be raised in rank among the sciences and in the estimation of the public. I hail the day when that which *has been* accomplished for astronomy and chemistry, when they advanced from astrology and alchemy, *shall be* accomplished for medicine! Then, and th[illegible]n only, will our profession be esteemed at its just value and [illegible]ain its just rank and dignity.

I be[illegible] add to this brief communication a remark on *two* points not [illegible] [illegible]onnected with its [illegible];

I understand that Dr. Horace Green, of New York, has cauterized the larynx successfully in epilepsy, passing through this orifice a spunge imbued with a solution of the nitrate of silver, attached to a piece of whalebone. It is plain that Dr. Green is impressed with the part that the larynx plays in this formidable malady. I am unable to form an idea of the degree of promise held out by the measure he has adopted. I trust I need not say how I shall rejoice if Dr. Green's efforts in the cause of science and of humanity prove successful in this instance.

But I have a confession to make. I was impressed with the idea of the difficulty attending the introduction of an instrument into or through the larynx. I still doubted, when Dr. Brainard stated that he had performed that operation; and I said let us try it on the dog. This was done by Dr. Brainard accordingly. The animal's mouth was held open, the tongue was drawn out, being held by the hand holding a dry towel, and the larynx was distinctly brought into view: the instrument was readily introduced *as* the larynx dilated during inspiration. That this was effected was further proved by passing the stomach tube: when this was inserted and a lamp brought near its opening, the flame was speedily extinguished.

The second point to which I have alluded, relates to tracheotony. Having explained my views to Dr. Brainard, that gentleman promptly acceded to my wishes to perform my operation on the dog.

The skin being divided, a pointed instrument, consisting of two blades, was made to pierce the trachea, and the blades were then separated; into the opening then made, a wire cage—made in the form of a tube, admitting of compression—was readily introduced, and the formidable operation was performed, occupying but a minute or two. The cage was of conical form and could not be readily made to enter further; it was so arranged as to *button* when within the orifice and could only be removed as it was introduced, by compressing the tube and so diminishing its diameter and size.

I think tracheotony need be scarcely more difficult or formidable than venesection. If so, in how many cases, will it tend to save life and intellect, whilst it modifies and mitigates *the* malady, emphatically denominated Herculean?

My own professional career is nearly terminated; therefore I may be allowed to call on my medical brethren everywhere, and now, especially in the United States, to aid in prosecuting an inquiry in which I have been for so many years engaged, not, I trust, altogether without benefit to my profession and to mankind.

